# On the road to C_4_ rice: advances and perspectives

**DOI:** 10.1111/tpj.14562

**Published:** 2019-11-14

**Authors:** Maria Ermakova, Florence R. Danila, Robert T. Furbank, Susanne von Caemmerer

**Affiliations:** ^1^ Australian Research Council Centre of Excellence for Translational Photosynthesis Division of Plant Science Research School of Biology The Australian National University Acton ACT 2601 Australia

**Keywords:** C4 photosynthesis, rice, metabolic engineering, bundle sheath cells, plasmodesmata, photosynthetic electron transfer

## Abstract

The international C_4_ rice consortium aims to introduce into rice a high capacity photosynthetic mechanism, the C_4_ pathway, to increase yield. The C_4_ pathway is characterised by a complex combination of biochemical and anatomical specialisation that ensures high CO_2_ partial pressure at RuBisCO sites in bundle sheath (BS) cells. Here we report an update of the progress of the C_4_ rice project. Since its inception in 2008 there has been an exponential growth in synthetic biology and molecular tools. Golden Gate cloning and synthetic promoter systems have facilitated gene building block approaches allowing multiple enzymes and metabolite transporters to be assembled and expressed from single gene constructs. Photosynthetic functionalisation of the BS in rice remains an important step and there has been some success overexpressing transcription factors in the cytokinin signalling network which influence chloroplast volume. The C_4_ rice project has rejuvenated the research interest in C_4_ photosynthesis. Comparative anatomical studies now point to critical features essential for the design. So far little attention has been paid to the energetics. C_4_ photosynthesis has a greater ATP requirement, which is met by increased cyclic electron transport in BS cells. We hypothesise that changes in energy statues may drive this increased capacity for cyclic electron flow without the need for further modification. Although increasing vein density will ultimately be necessary for high efficiency C_4_ rice, our modelling shows that small amounts of C_4_ photosynthesis introduced around existing veins could already provide benefits of increased photosynthesis on the road to C_4_ rice.

## Significance Statement

Installing a C_4_ CO_2_ concentrating mechanism into rice is arguably an ambitious molecular engineering initiative in plant science. This review maps the progress of the C_4_ rice project since its inception more than 10 years ago. Photosynthetic functionalisation of the bundle sheath compartment and manipulating leaf anatomy remain challenging but rapid progress in installing C_4_ biochemistry and manipulating multiple traits in a single genetic transformation has been made possible by advances in synthetic biology.

## Introduction

The C_4_ photosynthetic pathway is characterised by a complex combination of biochemical and anatomical specialisation, which provides an elevation of the CO_2_ partial pressure at the site of ribulose bisphosphate carboxylase oxygenase (RuBisCO) in leaf bundle sheath (BS) cells. CO_2_ is initially assimilated into C_4_ acids by phosphoenolpyruvate (PEP) carboxylase in mesophyll (M) cells. These acids then diffuse to and are decarboxylated in BS cells in which CO_2_ is concentrated. This spatial separation of initial CO_2_ fixation in M cells and subsequent refixation by RuBisCO in BS cells is mostly associated with Kranz anatomy, but can also occur in single cells (Bowes and Salvucci, 1984; Voznesenskaya *et al.*, 2001). C_4_ photosynthesis has evolved independently more than 60 times, providing one of the most widespread and effective solutions for overcoming the catalytic inefficiency of RuBisCO (Sage *et al.*, 2012; Christin and Osborne, 2013). Because of the superior nitrogen and water use efficiency of C_4_ plants, transfer of C_4_ photosynthetic traits has been an early strategy for improving C_3_ photosynthesis (Sage, 2004; Miyao *et al.*, 2011). Initial attempts were made to generate hybrids between closely related C_3_ and C_4_ plants by conventional crossing (reviewed in Brown and Bouton, 1993). With the advent of transformation technology there were numerous attempts to introduce C_4_ photosynthetic genes into C_3_ species (reviewed in Matsuoka *et al.*, 2001; Hausler *et al.*, 2002; Miyao, 2003). These all sought to insert a C_4_ like pathway into M cells of C_3_ species following the blue print of the aquatic single cell organism *Hydrilla verticillata* (Bowes and Salvucci, 1984; von Caemmerer *et al.*, 2014). Four enzymes of the C_4_ photosynthetic pathway were successfully introduced into rice by Miyao and collaborators and although no photosynthetic gains were observed, Miyao *et al. *(2011) have elegantly summarised the extensive contribution this endeavour made to our understanding of gene expression and regulation of C_4_ genes expressed in rice. Current progress on building a two cell C_4_ pathway in rice are built on these earlier insights.

The current C_4_ rice project (https://c4rice.com/) which aims to introduce Kranz anatomy into rice was first conceived by John Sheehy (Mitchell and Sheehy, 2006) who invited a group of C_4_ photosynthesis experts to the international rice research institute (IRRI) in the Philippines to discuss the potential of C_4_ rice. Engineering the C_4_ pathway into a C_3_ plant requires manipulation of both anatomical and biochemical traits and progress of this research has been reviewed a number of times (Hibberd *et al.*, 2008; Hibberd and Covshoff, 2010; Langdale, 2011; Sedelnikova *et al.*, 2018). To introduce Kranz anatomy into rice requires a change of vein spacing patterns so that veins are closer together in the leaf and BS cells need to be ‘functionalised’ for increased photosynthetic capacity, including increased chloroplast content. At this point while there are established candidates for the genes and transcription factors that potentially control vein spacing, the complete transcriptional network remains to be elucidated (for review see Sedelnikova *et al.*, 2018). However, some progress has been made in photosynthetic functionalisation of the rice BS (Wang *et al.*, 2017b) While insertion of C_4_ biochemistry in rice faces challenges of engineering high level and cell‐specific gene expression (Hibberd and Covshoff, 2010), genes encoding the C_4_ pathway enzymes and most of the metabolite transporters have now been identified. Here we review current progress and how they have been enabled by technological advances in cloning techniques and highlight future challenges.

## Building the biochemistry for C_4_ rice

**Figure 1:**
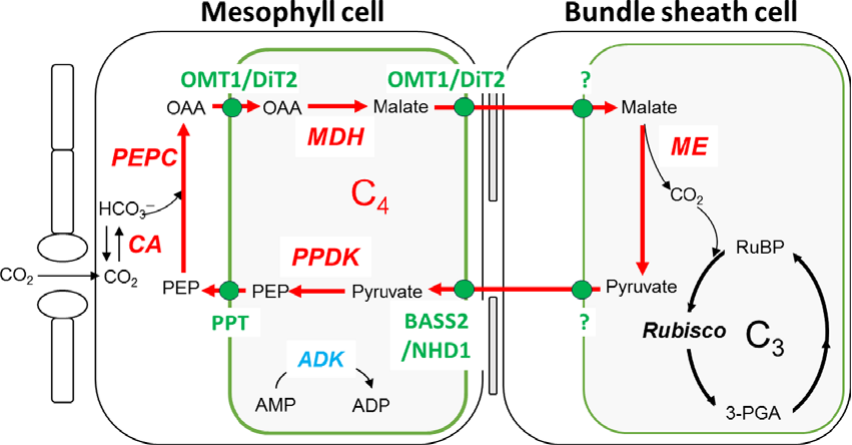
Enzymes and transporters included in the construction of C_4_ rice. These include carbonic anhydrase (CA), PEP carboxylase (PEPC), malate dehydrogenase (MDH), NADP‐malic enzyme (ME), and pyruvate Pi dikinase (PPDK). Adenylate kinase (ADK) has been added to ensure AMP is converted to ADP. The required transporters are oxoglutarate/malate transporter (OMT1), PEP/phosphate translocator (PPT), dicarboxylate transporter (DiT2), pyruvate/sodium symporter (BASS2), and sodium/proton antiporter (NHD1). Malate importer and pyruvate exporter in bundle sheath chloroplasts are not yet confirmed. Other abbreviations used are OAA for oxaloacetate and PEP for phosphoenolpyruvate.

In 2008, the year the C_4_ rice consortium commenced, a remarkable amount of information in regard to the genes encoding the key proteins in the C_4_ pathway was already known. The cDNA sequences for maize phosphoenolpyruvate carboxylase (PEPC), pyruvate orthophosphate dikinase (PPDK), and NADP‐malate dehydrogenase (MDH) had all been reported and expressed in the M of cells of rice (reviewed in Miyao *et al.*, 2011). The cDNA sequence for NADP‐malic enzyme (NADP‐ME) had also been reported for both the rice endogenous gene and the maize gene (Drincovich *et al.*, 2001). The genes encoding carbonic anhydrase (CA) in maize had also been cloned by this time, but the precise identity of the gene product located in the cytosol of the M cells in C_4_ leaves was not definitively proven (reviewed in DiMario *et al.*, 2016). Therefore, the key genes encoding the entire biochemical pathway of the NADP‐ME type C_4_ mechanism as shown in Figure 1 were all available at the commencement of the C_4_ rice project.

What set the current C_4_ rice project apart from previous activities was the desire to engineer a full Kranz two cell‐type mechanism in rice (von Caemmerer *et al.*, 2012). This approach requires not only the cDNA sequences for the relevant photosynthetic proteins but high level expression with suitable promoters in the appropriate cell type of rice. For M cell‐specific expression, the promoters of genes from C_4_ species encoding PEPC, PPDK, and aspartate aminotransferase (AspAT) had all been tested in C_3_ species and lead to M‐specific accumulation of the β glucuronidase (GUS) reporter protein (Hibberd and Covshoff, 2010). The PEPC promoter from maize had been the most extensively tested in rice and various truncated versions have been shown to produce M‐specific expression of the reporter gene GUS in rice (Hibberd and Covshoff, 2010).

High level BS cell‐specific expression of proteins such as NADP‐ME has proven to be the greatest challenge for establishing a C_4_ metabolic pathway in rice. While there are potential anatomical constraints in regard to the photosynthetic competence of the rice BS compartment (Wang *et al.*, 2017b), the paucity of promoters available to drive BS expression in a C_3_ plant has been a major obstacle. At the commencement of the C_4_ rice project, the promoters of the genes encoding phosphoenolpyruvate carboxykinase (PEPCK), NADP‐ME, AspAT, small subunit (Engelmann *et al.*) of RuBisCO, and the P subunit of glycine decarboxylase (GDCP) from C_4_ species had been tested in C_3_ plants (Chen *et al.*, 2001; Nomura *et al.*, 2005a; Nomura *et al.*, 2005b; Engelmann *et al.*, 2008). Of these, a version of the *Zoysia japonica* PEPCK promoter (Nomura *et al.*, 2005a) resulted in BS‐specific GUS accumulation in rice. The *Flaveria trinervia* GDCP promoter was shown to be BS/ vascular‐specific in Arabidopsis and the promoter of this gene from *Flaveria anomala* (Chen *et al.*, 2001) was vascular‐specific in rice. Both the PEPCK and GDCP promoters have subsequently been utilised in the C_4_ rice strategy although promoter strength has been an ongoing issue for engineering efforts and it is notable that in the single cell C_4_ project (reviewed in Miyao *et al.*, 2011), genomic clones often but not always gave superior expression levels compared with cDNAs driven by their own promoters.

Stacking of genes in transgenic rice in the initial phases of this project required the crossing of homozygous lines harbouring single gene constructs to build a rice plant expressing a complete set of the genes encoding the major enzymes of the C_4_ pathway. Such a crossing strategy was a hugely time consuming effort as expression levels and cell‐specific expression of the recombinant protein must be checked for each line and a crossing donor identified for each transgene. With segregation of the transgenes inserted at different loci in the stacked lines, many hundreds of individuals had to be genotyped at each cross to obtain a single line harbouring the genes encoding the five key photosynthetic enzymes shown in Figure 1. Indeed, this crossing strategy has taken almost 6 years to achieve in *indica* rice in the current project. However, five genes are not enough for creating C_4_ rice and the biochemical pathway is being complemented by a suite of membrane transporters ensuring fast transport of metabolites between cell compartments. Most of the transporters have now been identified and are listed in Figure 1, however there is still some uncertainty about malate import to the BS chloroplast and the export of pyruvate following malate decarboxylation. A recent review of transporters was given by Schuler *et al. *(2016). Physiological gas exchange techniques exist to allow for the identification of a complete, functioning C_4_ pathway. These include measurements of CO_2_ compensation points, reduced oxygen sensitivity of CO_2_ assimilation and reduced carbon isotope discrimination (Furbank *et al.*, 2009). In the meantime new ^13^C pulse chase labelling techniques have been developed to analyse the paths of carbon during C_3_ and C_4_ photosynthesis (Arrivault *et al.*, 2009) and confirmed that malate is in fact being formed in our current five gene rice transgenics.

## Synthetic biology accelerates the pace

A consensus definition drafted by a group of European experts more than a decade ago defined synthetic biology as follows: ‘Synthetic biology is the engineering of biology: the synthesis of complex, biologically based (or inspired) systems, which display functions that do not exist in nature. This engineering perspective may be applied at all levels of the hierarchy of biological structures—from individual molecules to whole cells, tissues and organisms. In essence, synthetic biology will enable the design of ‘biological systems’ in a rational and systematic way’ (Serrano, 2007). Synthetic biology has evolved and adopted many of the commonly used terms in mainstream engineering such as ‘switch’, ‘rewire’, ‘design, test and redesign cycle’. Although in the creation of C_4_ rice, in which a template or design already existing in nature is being used, the installation of up to 20 genes to completely ‘rewire’ rice metabolism and anatomy surely fits the definition above. A major limitation in the synthetic biology approach however is the cycle time for the design, test and redesign in crops such as rice.

The last 5 years have seen an exponential growth in synthetic biology tools and the cost of gene synthesis has plummeted. This has enabled the C_4_ rice consortium to adopt a more rapid cycle of design, test and prototype coupled to the adoption of a rapid *Agrobacterium*‐based rice transformation system in the *japonica* rice variety ‘Kitaake’. Kitaake is fast flowering, day neutral, small in stature and an established model for functional genomics studies (Li *et al.*, 2017). The obstacle of genetic transformation with a single gene construct at a time and crossing has largely been solved by gene synthesis and Golden Gate cloning or similar ‘gene building block’ approaches (Engler *et al.*, 2014). Gene synthesis allows the ‘domestication’ of coding sequences to remove or insert rare Type IIS restriction enzyme recognition sites while leaving the amino acid sequence unchanged, thus enabling the assembly of gene modules which can be pasted together, often in a ‘one pot cloning’ approach. Assembly of these modules into a T‐DNA suitable for *Agrobacterium* transformation is therefore greatly accelerated over traditional restriction/ligation approaches (Andreou and Nakayama, 2018). In principle, assembling all the metabolic and transporter components of Figure 1 for rice transformation on a single construct should be readily achievable and this work is currently underway. This approach has so far enabled a 6‐year crossing strategy to be reduced to a 6‐month single transformation experiment in rice.

In a large multigene overexpression construct, it is not desirable to reuse ‘parts’ multiple times due to the possibility of recombination deletion, post‐transcriptional gene silencing or inactivation at the promoter level via methylation (Wassenegger, 2002). Epigenetic promoter silencing is a poorly understood process and can be a major challenge for metabolic engineering. This also presents a challenge for the design of the gene constructs described above for C_4_ rice. For example, expression of just CA, PEPC, MDH and PPDK would ideally require four heterologous M‐specific promoter sequences. While this may be possible for the M compartment, it is not for the BS compartment (see above).

Synthetic biology has also provided a potential solution to this paucity of promoters in rice. Brückner *et al. *(2015) described a system compatible with the Golden Gate cloning which utilises multiple promoters (Synthetic TALE Activated Promoters or STAPs) designed to be orthogonal to the genome of the plant to be transformed, which can be activated by a single Transcription Activator‐Like Effector (TALE). This approach provides the opportunity to build multiple transcriptional units driven by different promoters on the same gene construct, *trans*‐activated by a single transcription factor.

## Functionalisation of the bundle sheath: an anatomical roadblock?

**Figure 2:**
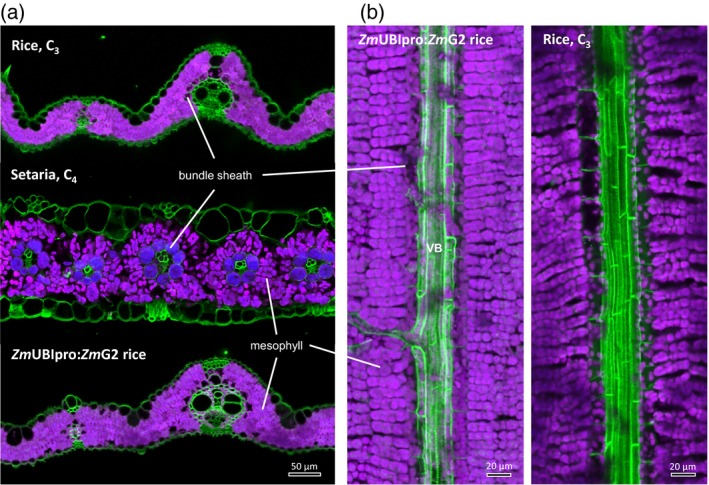
Confocal micrographs of fresh hand‐cut transverse (a) and paradermal (b) leaf sections of *Oryza sativa* (rice), *Setaria viridis* (Setaria), and a rice line with constitutively expressed GOLDEN2 transcription factor from *Zea mays* (*Zm*UBIpro:*Zm*G2 rice). Excitation wavelength at 633 nm and dual emission wavelengths at 650–720 nm (magenta for Photosystem II) and 720–800 nm (blue for Photosystem I) were used to visualise chloroplasts. Note that bundle sheath cells of *Setaria* leaf have chloroplasts appearing pseudo‐blue due to reduced Photosystem II content. Cell wall (green) was visualised at an excitation wavelength of 405 nm and emission wavelength of 420–480 nm. VB, vascular bundle.

**Figure 3:**
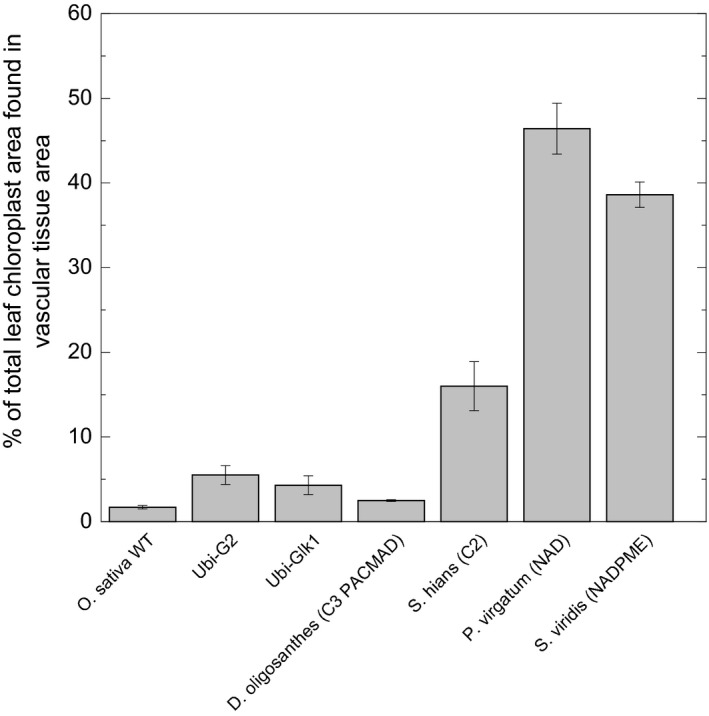
Per cent of total chloroplast area found in vascular tissue area. The data are taken from Table S5 Wang et al. (2017b). Ubi‐G2 and Ubi‐Glk1 are *Oryza sativa* plants in which the transcription factor GOLDEN2 (G2) or GOLDEN2‐LIKE1 (GLK1) has been overexpressed from the ubiquitin promoter of *Zea mays* (Wang et al., 2017b). Other monocots shown are *Dichanthelium oligosanthes*, a C_3_ from the PACMAD clade; *Steinchisma hians,* which operates the C_2_ photosynthetic pathway; *Panicum virgatum,* a C_4_ species with the NAD‐ME decarboxylation type; and *Setaria viridis*, a C_4_ species with the NADP‐ME decarboxylation type.

Figure 2(a) shows fresh transverse sections of a rice (C_3_) and *Setaria viridis* (C_4_) leaf imaged with the laser confocal microscope using chlorophyll fluorescence overlaid with cell wall fluorescence. This enables visualisation of the chloroplast contents of BS cells in each species. This image clearly indicates that the BS compartment of the C_4_ leaf is tightly packed with chloroplasts, whereas the rice BS compartment is only sparsely populated by chloroplasts and the cells are highly vacuolated. This has previously been pointed out and ‘photosynthetic functionalisation’ of the BS has been proposed as an early step in evolution of C_4_ photosynthesis, probably occurring at the C_2_ or proto‐Kranz stage (Sage, 2004; Wang *et al.*, 2017b). The underlying mechanisms responsible for the proliferation of chloroplasts in BS cells of C_4_ leaves are largely unknown. However, recently a transgenic approach has been used in rice to ‘recreate’ this key step in evolution by overexpressing the transcription factors GOLDEN2 (G2) or GOLDEN2‐LIKE (GLK) (Wang *et al.*, 2017b) which had been implicated as important in BS cell differentiation in terrestrial plants including *Zea mays* and more recently in rice (Wang *et al.*, 2013 and references therein). These transcription factors are thought to act in the cytokinin signalling pathway (Wang *et al.*, 2013). Overexpression of these *Z. mays* transcription factors in rice indeed increased the proportion of vascular cell area occupied by chloroplasts, the mitochondrial population in rice BS cells, and the plasmodesmatal connectivity at the BS/M cell interface (Wang *et al.*, 2017b). The BS chloroplast abundance data from this work are summarised in Figure 3. It is evident from these quantitative data that overexpression of this class of transcription factor can increase BS chloroplast content as a proportion of total leaf chloroplasts by approximately five‐fold (in the case of G2 expressed from the ubiquitin promoter of *Z. mays*: UBI‐G2). However, another three‐fold increase is required to reach levels equivalent to C_2_ leaves and potentially another six‐fold to reach C_4_ levels of chloroplast area in the BS. This can be visualised in Figure 2 which shows a representative image of a leaf transverse (a) and paradermal (b) sections of the UBI‐G2 lines (Wang *et al.*, 2017b) compared with rice and Setaria leaves. Comparison of rice and C_4_ leaf chloroplast distribution is complicated, however, by the change in vein spacing and reduction in M chloroplast numbers seen in C_4_ leaves relative to C_3_ (Stata *et al.*, 2016).

Alternative approaches are also under investigation for increasing chloroplast abundance/ volume in the BS compartment of rice (Wang *et al.*, 2017a). The transcription factor CYTOKININ RESPONSIVE GATA FACTOR 1 (CGA1) has also been shown to regulate leaf chloroplast abundance via the cytokinin signalling network (Chiang *et al.*, 2012; Hudson *et al.*, 2013; Wang *et al.*, 2017a) and overexpression of CGA1 in rice resulted in a 30% increase in flag leaf chlorophyll and close to a doubling of chloroplast numbers on a fresh weight basis (Hudson *et al.*, 2013). It has been proposed that regulation of the FtsZ chloroplast division gene by CGA1 is responsible for this phenotype, presenting opportunities for regulating levels of this protein directly or other genes in this pathway controlling chloroplast division (Hudson *et al.*, 2013; Wang *et al.*, 2017a).

It may be that part of the difficulty obtaining high level expression of chloroplast proteins in rice BS cells is the lack of sufficient chloroplast volume to house the recombinant proteins targeted to this compartment. The developmental programme of BS cells in a C_3_ grass may reflect a more ‘parenchyma‐like’ role in temporary sugar storage and the sugar status of the BS cells may not be conducive to photosynthetic functionalisation. The high level expression of sugar effluxers such as the SWEET13 gene family in the BS of C_4_ plants has been proposed as evidence that sugar status in the BS or C_4_ grasses may be quite different from that in C_3_ grasses (Emms *et al.*, 2016). In addition, partitioning of starch almost exclusively into the BS of C_4_ grasses is a curious and potentially relevant observation (Lunn and Furbank, 1999).

## What can we learn from comparative leaf anatomy between C_3_ and C_4_ grass species?

**Figure 4:**
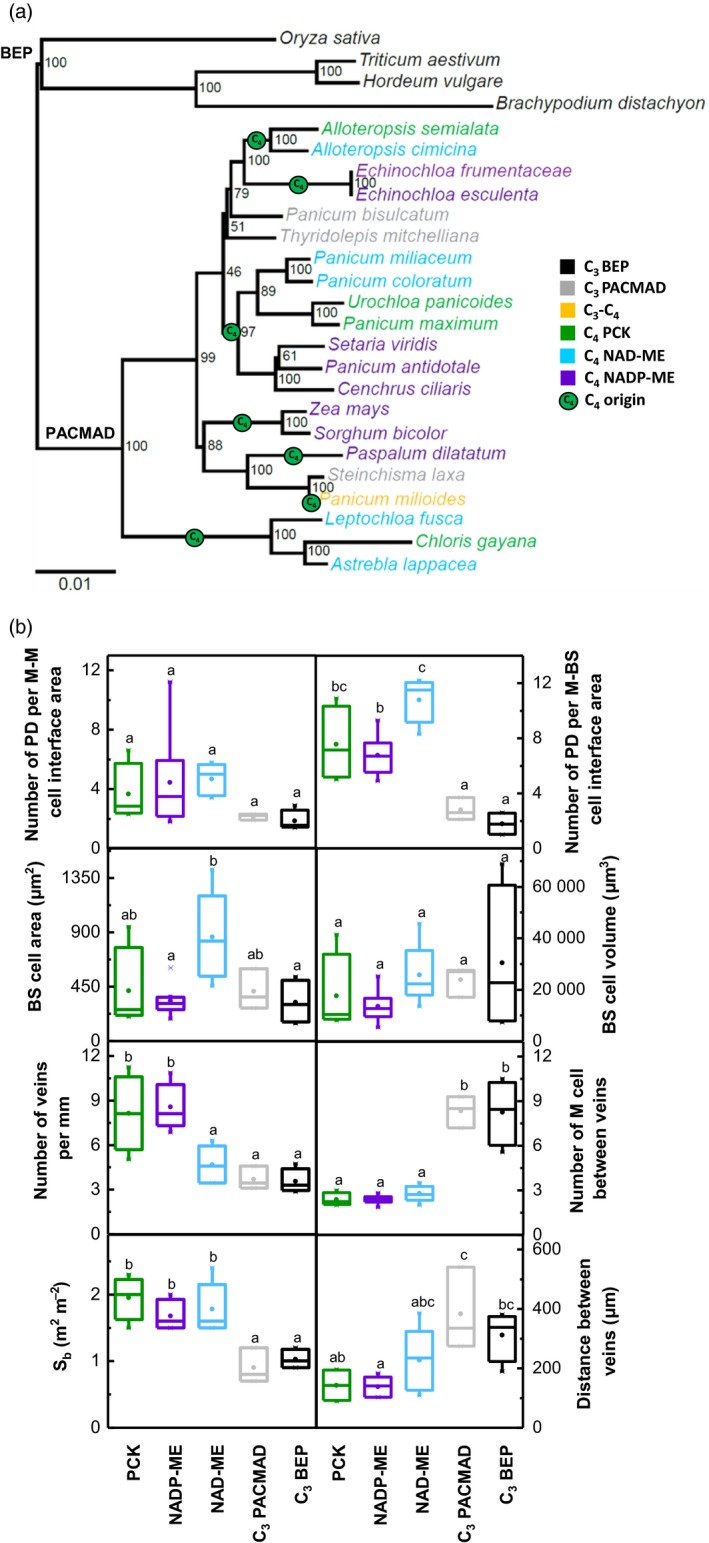
(a) Phylogenetic tree of the C_3_ and C_4_ grass species examined generated using sequences from ndhF and rbcL chloroplast genes. Species names are colour‐coded according to photosynthetic types. The seven independent evolutionary origins of C_4_ photosynthesis according to (GPWGII, 2012) are indicated with green circles at the midpoint of the branches. Note that *Panicum milioides*, a.k.a. *Steinchisma hians*, although technically a C_3_–C_4_ intermediate (Duvall *et al.*, 2003) was categorised as C_4_ in GPWGII (2012). Numerical value at internal nodes is the percentage of non‐parametric bootstrap replicates that support the bipartition. Scale bar indicates amino acid substitutions per site. (b) Distribution of leaf trait values among photosynthetic types in (a) excluding the C_3_–C_4_ intermediate type, in which only one species was measured. The distribution of eight variables is summarised by boxplots. Box and whiskers represent the 25 to 75 percentile, and the minimum and maximum distribution. Means are denoted by (•). Letters show the statistical ranking using a post‐hoc Tukey test among photosynthetic types (different letters indicate differences at *P‐*value < 0.05). BS, bundle sheath; M, mesophyll; PD, plasmodesmata; S_b_, bundle sheath surface area per unit leaf area.

It has previously been proposed that an early step in C_4_ evolution was ‘inflation’ of BS cell size (Sage, 2004; Sage *et al.*, 2012; Christin *et al.*, 2013). However, our results from anatomical measurements performed in 25 grass species, representing different photosynthetic types and seven independent C_4_ evolutionary origins (Figure 4a), reveal that C_4_ leaves do not necessarily have larger BS cells, nor do they always have shorter interveinal distances than C_3_ leaves (Figure 4b). Rather, a C_4_ leaf has greater BS surface area per leaf area (S_b_), fewer interveinal M cells than a C_3_ leaf and most notably, more plasmodesmata (PD) at the BS/M cell interfaces (Figure 4; Danila *et al.*, 2016; Danila *et al.*, 2018). For a functional C_4_ rice, these findings mean that increasing PD connections between M and BS, increasing S_b_, and reducing M cells between veins of the rice leaves may be essential for the operation of an efficient photosynthetic pathway.

It has long been thought that a key feature of C_4_ leaf anatomy was increased abundance of the symplastic nanochannels (PDs) that facilitate the rapid exchange of metabolites between M and BS cells during C_4_ photosynthesis (Hatch and Osmond, 1976). Recent work, however, has provided quantitative data on this parameter (Danila *et al.*, 2016; Danila *et al.*, 2018); Figure 4). Enhancement of the PD connections between M and BS of rice to levels observed in closely related C_4_ species would require increases of at least five‐fold (Danila *et al.*, 2018). While there are few genes known which control PD development and proliferation, it was recently observed that when maize GLK genes were constitutively expressed in rice, increased organelle volume was accompanied by increased M‐BS PD density; although the enhancement is only double that of wild type (Wang *et al.*, 2017b). Coordination of chloroplast and PD development has been suggested previously (Brunkard *et al.*, 2013) and cytokinin has also been implicated in the proliferation of PDs in the shoot apical meristem (Ormenese *et al.*, 2006). These findings offer some hope that a single gene or at least an established transcriptional network could provide a master switch for C_4_ BS anatomy.

The BS surface area per leaf area (S_b_) is a physiological parameter which has proven to be an important feature of modelling the M‐BS interface, including to estimate BS conductance to CO_2_ diffusion (first estimates ranged between 0.6 m^2^ m^−2^ and 3.1 m^2^ m^−2^ (Apel and Peisker, 1978; Brown and Byrd, 1993)). S_b_ was obtained by dividing measurements of BS tissue perimeter from micrographs by interveinal distance (Pengelly *et al.*, 2010). Increasing S_b_ can be achieved in multiple ways; modification of BS cell size, vasculature size, and the distance between BS. Because BS cell size does not differ substantially between C_3_ and C_4_ grass species (Figure 4, Danila *et al.*, 2018), reducing the interveinal distance appears to be the most logical path to increase S_b_ in rice. Ideally, this would mean upregulation of gene(s) that would promote insertion of additional veins between existing veins of rice, thus reducing interveinal distance and, at the same time, decreasing the number of M cells between veins (Sedelnikova *et al.*, 2018; Hughes *et al.*, 2019).

## Paying the energy cost of C_4_ photosynthesis

The energy cost of C_4_ photosynthesis is significantly higher compared with C_3_ as it requires a minimum of two more ATP molecules per one CO_2_ fixed (Furbank *et al.*, 1990). While high conductance of the M–BS interface to metabolites is essential for the operation of C_4_ photosynthesis, it also allows a proportion of the CO_2_ concentrated in the BS to escape back to M (called CO_2_ leakage; von Caemmerer and Furbank, 2003). This CO_2_ is either refixed or lost to the intercellular spaces in the M, which increases the cost of C_4_ photosynthesis (Furbank *et al.*, 1990; von Caemmerer and Furbank, 2003). To sustain higher energy requirements, C_4_ plants adapt the photosynthetic electron transfer chains of M and BS cells depending on specific needs of the C_4_ subtype they belong to (Munekage and Taniguchi, 2016). As the efforts of the C_4_ rice project have targeted NADP‐ME as the decarboxylating enzyme, we focus here on specific energy requirements of the NAPD‐ME subtype of C_4_ photosynthesis.

**Figure 5:**
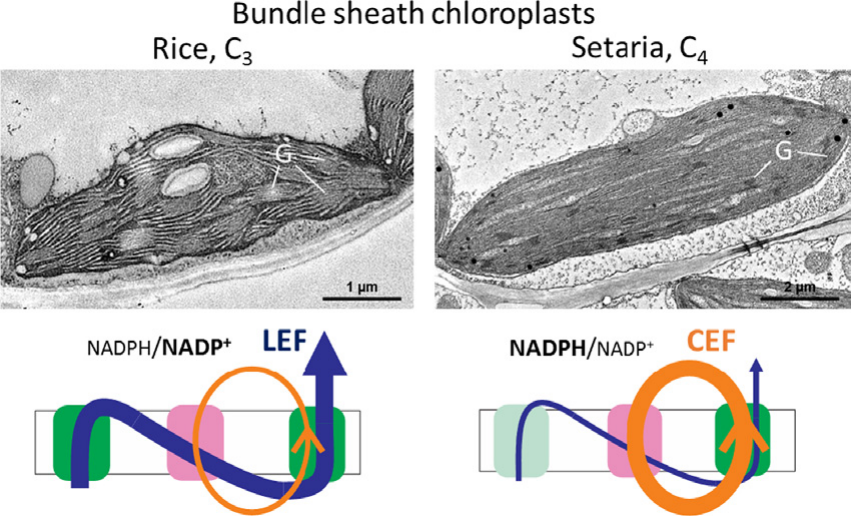
Transmission electron micrographs of the bundle sheath chloroplasts from *Oryza sativa* (rice) and *Setaria viridis* (Setaria) and schematic representation of their electron flow pathways. LEF, linear electron flow, is supported by Photosystem II, Cytochrome *b*_6_ *f* complex and Photosystem I and is dominant in rice bundle sheath chloroplasts. CEF, cyclic electron flow, does not require Photosystem II and is dominant in Setaria bundle sheath chloroplasts. G, thylakoid grana stacks, are abundant in rice but not in Setaria because bundle sheath chloroplasts of NADP‐ME C_4_ plants require less Photosystem II that is typically localised to grana. NADPH/NADP^+^ balance in chloroplasts might be a factor defining the ratio between LEF and CEF.

An early observation on the anatomy of tropical grasses which predated the discovery of C_4_ photosynthesis was that chloroplasts in the two cell types of leaves with Kranz anatomy are dimorphic, with the BS chloroplasts often lacking grana stacks (Rhoades and Carvalho, 1944). Subsequently, it was discovered that these grasses such as *Z. mays* were in fact C_4_ plants and specifically used the NADP‐ME pathway of C_4_ photosynthesis (Edwards *et al.*, 1971; Hatch and Kagawa, 1976). As NADP‐ME plants primarily use malate as a C_4_ acid diffusing from M to BS cells, NADPH produced in the M chloroplasts is consumed for malate synthesis but it is then produced in the BS upon malate decarboxylation to pyruvate (Figure 1). The net transfer of NADPH from M to BS cells means that there is a reduced requirement for linear electron flow to produce NADPH in the BS chloroplast, which have reduced Photosystem II (PSII) content but have highly developed cyclic electron flow (CEF) machinery around Photosystem I (PSI) (Figure 5). Therefore, the observation that BS chloroplasts of NADP‐ME plants are mostly agranal with little or no grana thylakoids functionally reflects the reduced PSII, which would normally be located in the granal stacks (see Munekage and Taniguchi, 2016). However, grana content in BS chloroplasts of NADP‐ME plants is rather variable between species (Ueno *et al.*, 2005) and also within species in response to environmental conditions (Omoto *et al.*, 2009; Danila *et al.*, 2019) and depending on leaf age (Andersen *et al.*, 1972). It has also been shown that aspartate can be used as a transported C_4_ acid in many NADP‐ME type C_4_ plants, providing some flexibility in the amount of reducing power transferred to the BS from the M and suggesting that PSII content in the BS cells might in fact respond to the NADPH/NADP^+^ ratio or the redox state of the BS cells (see Furbank, 2011). This is supported by overexpression of maize NADP‐ME in rice M cells which resulted in agranal chloroplasts, conceivably, due to the increased NADPH/NADP^+^ ratio depleting PSI of electron acceptor and causing over‐reduction of the electron transfer chain (Takeuchi *et al.*, 2000). While prolonged reduction of PSII acceptors promotes the formation of reactive oxygen species and causes damage and degradation of PSII (Vass, 2012) and thus also grana, there might be also regulatory mechanisms preventing transcription and *de novo* assembly of PSII polypeptides and grana formation in over‐reduced conditions (Pfannschmidt *et al.*, 1999).

It has been proposed that elevated CEF in BS chloroplasts allows NADP‐ME plants to accommodate the extra costs of C_4_ photosynthesis by producing ATP without affecting NADPH/NADP^+^ ratio (Furbank *et al.*, 1990). The chloroplast NADPH dehydrogenase‐like complex and PROTON GRADIENT REGULATION 5 (PGR5) mediate two different CEF routes (Takabayashi *et al.*, 2005; Munekage, 2016). Interestingly, in C_3_ plants, CEF is promoted in conditions causing over‐reduction of the electron transport chain (Suorsa, 2015) and therefore CEF might be naturally upregulated in BS cells of C_4_ rice in response to high NADPH/NADP^+^ ratio.

At present, it is unknown whether alteration of BS chloroplast electron transport components is a strict requirement for C_4_ rice or only ‘fine tuning’. If the required regulatory mechanisms already exist in rice, BS chloroplasts could conceivably adjust PSII and grana content according to the redox state of cells in C_4_ rice (Figure 5). Consequently, just the right amount of PSII will be fully assembled in BS to donate electrons for the CEF and compensate for the shortage of NADPH via linear electron transfer. However, the desired adaptation of electron transport requires a strictly coordinated expression and activity of NADP‐ME in rice BS cells and efficient NADPH oxidation by the Calvin cycle to maintain an appropriate NADPH/NADP^+^ balance. It is worth mentioning that the flexibility of PGA reduction between M and BS cells in C_4_ plants also contributes to the maintenance of NADPH/NADP^+^ balance in BS cells, however, it is not clear whether this pathway will be immediately available in C_4_ rice. The composition of thylakoid protein complexes and energy requirements are similar between C_3_ and C_4_ M cells (Munekage and Taniguchi, 2016), but rice BS chloroplasts will require some reorganisation of thylakoid complexes and increased abundance of the NADPH dehydrogenase‐like complex (Majeran *et al.*, 2008; Hernández‐Prieto *et al.*, 2019). This fine tuning will be necessary to run an efficient C_4_ pathway as our recent research shows that the rate of C_4_ photosynthesis is strongly dependent on the electron transport capacity of both M and BS cells (Ermakova *et al.*, 2019).

## Partial C_4_: modelling mixed C_3_ and C_4_ photosynthesis on the path to C_4_ rice

The concept of C_4_ rice has excited photosynthetic modellers and there are a number of photosynthetic models that have tried to evaluate the efficacy of introducing C_4_ photosynthesis into rice (Bellasio, 2016; Yin and Struik, 2017; Wang *et al.*, 2017c; Bellasio and Farquhar, 2019). Each of these models has a different focus. Wang *et al.* (2017c) developed a 3D reaction diffusion model of BS and connected M cells with anatomy based on a C_3_ rice leaf in which C_4_ photosynthesis was integrated with existing C_3_ photosynthesis. They concluded that the C_4_ cycle can operate adjacent to C_3_ photosynthesis in rice M cells, but that the energy partitioning between the C_3_ and C_4_ cycle is an important consideration. In their current model every M cell is adjacent to a BS cell so it is built on a rice leaf with altered vein spacing. Bellasio’s models consider C_4_ photosynthesis as an addition to C_2_ photosynthesis and also provides a valuable discussion on energy partitioning. Yin and Struik (2017) consider C_4_ photosynthesis in rice at the canopy and crop level in different environments.

**Figure 6:**
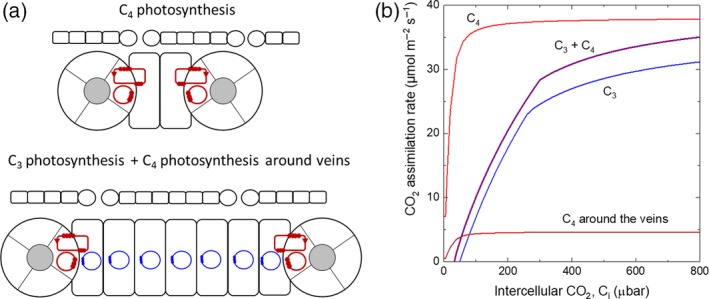
(a) Diagram comparing two cell‐type C_4_ photosynthesis and a rice leaf with low capacity C_4_ photosynthesis occurring around the veins. In the latter, C_3_ photosynthesis occurs in all mesophyll cells (rectangular cells) and in bundle sheath cells (cells inside circles), in addition to C_4_ photosynthesis occurring only in mesophyll cells adjacent to bundle sheath cells. (b) Modelled CO_2_ assimilation rates of a rice leaf with low capacity C_4_ photosynthesis occurring around the veins (purple line). Also shown are rice leaf with C_3_ photosynthesis (blue line), a standard high capacity C_4_ photosynthesis (red line), and the C_4_ photosynthesis around the vein contributing to the enhanced photosynthesis rate of the purple line (dark red line). C_3_ leaf photosynthesis has been modelled with RuBisCO maximal activity, V_cmax_ = 90 µmol m^−2^ sec^−1^ and electron transport rate, J = 150 µmol m^−2^ sec^−1^. The standard C_4_ photosynthesis was modelled with V_cmax_ = 40 µmol m^−2^ sec^−1^ and PEP carboxylase maximal activity, V_pmax_ = 300 µmol m^−2^ sec^−1^. The low capacity C_4_ photosynthesis around the veins was modelled with V_cmax_ = 5 µmol m^−2^ sec^−1^ and V_pmax_ = 25 µmol m^−2^ sec^−1^. In the combined C_3_ and C_4_ photosynthesis, the total V_cmax_ was maintained at 90 µmol m^−2^ sec^−1^. C_3_ RuBisCO kinetic constants by von Caemmerer (2000) were used together with the Michaels constant for CO_2_ of PEPC of 154 µbar (Boyd *et al.*, 2015).

It is common to compare rice with *Z. mays* but it is informative to widen this comparison. The analysis of 25 monocot C_3_ and C_4_ leaves shows that all C_4_ leaves have closer vein spacing than C_3_ species with at most 2–3 M cells between BS (Figure 4). However there are examples in which C_4_ photosynthesis is naturally supported around widely spaced veins such as in maize husk tissue, albeit at lower rates with little photosynthetic activity in the interveinal M cells (Langdale *et al.*, 1988; Pengelly *et al.*, 2011). Here, in the context of the introduction of C_4_ metabolism into rice without altered vein spacing, we have asked the simple question: can we detect a small amount of C_4_ photosynthesis introduced around existing veins using gas exchange techniques? The modelling here uses the Farquhar *et al. *(1980) C_3_ model of photosynthesis combined with the enzyme limited C_4_ model of photosynthesis described in von Caemmerer (2000) with C_3_ kinetic constants for RuBisCO. Rice leaves have approximately 7 M cells between veins (Figure 4b; Chatterjee *et al.*, 2016). The modelled curves show that partitioning a small amount of RuBisCO to low capacity C_4_ photosynthesis around veins (i.e. in 2 out of 7 M cells) lowers the compensation point and increase CO_2_ assimilation rates at all CO_2_ partial pressures (Figure 6). Hence this should be easily detected with gas exchange techniques. The modelling approach is very basic and has not considered the added energy requirements in M cells running both C_3_ and C_4_ photosynthesis given the low capacity C_4_ photosynthetic rates considered here. Nevertheless it suggests that even this small addition, which would result in the small amount of RuBisCO in BS being more efficient, could have a physiological benefit.

## Conclusions

Considerable progress has been made in building C_4_ rice. The initial concept of assembling a tool box of components and a biochemical, anatomical and molecular blueprint based on evolution and 50 years of study has now become one of the largest synthetic biology projects of all time in plant biology. Discovering that our ‘toolbox’ was often lacking and that new knowledge and technologies would continually reshape the strategy and redefine the blueprint should come as no surprise. ‘Functionalisation’ of the BS compartment is the immediate hurdle. For a fully functional C_4_ cycle to operate in rice across two cell types, the BS must be functionally capable of housing the photosynthetic machinery necessary for C_4_ acid decarboxylation and CO_2_ refixation by RuBisCO. It appears that we have the biochemical and molecular‐genetic components necessary for C_4_ function; the means to prototype and fine tune them; a promise that C_4_ photosynthesis around rice leaf veins may be possible and even beneficial, but there is still some distance yet to be travelled on the road to C_4_ rice.

## Author Contributions

All authors contributed to the writing of this review.

## Conflict of Interest

The authors declare no conflict of interest.

## Data Availability

The datasets generated in this paper are available from the corresponding author on request.
